# Development and validation of self-assessment instrument to measure the digital professionalism of healthcare professionals using social media

**DOI:** 10.1186/s12909-024-05142-6

**Published:** 2024-03-06

**Authors:** Shazia Imran, Rahila Yasmeen, Memoona Mansoor

**Affiliations:** 1 Department of Anatomy, NUST School of Health Sciences (NSHS), National University of Science and Technology, Islamabad, Pakistan; 2 Dean Riphah Academy of Research & Education, Riphah International University, Islamabad, Pakistan; 3 Department of Medical Education, Islamabad Medical & Dental College, Shaheed Zulfiqar Ali Bhutto Medical University, Islamabad, Pakistan

**Keywords:** Social media, Digital professionalism, Healthcare professionals, Validity, Reliability

## Abstract

**Background:**

The use of social media across the globe has risen incrementally. During the COVID-19 pandemic, these sites undeniably provided new avenues for professional networking but also led to a surge in cases of online misconduct. Professionalism instruments and scales do not assess the digital attitude and behaviour of healthcare professionals (HCPs). The purpose of this study was to identify the domains and items of digital professionalism related to social media use and to validate a self-assessment instrument to assess the digital professionalism of HCPs using social media.

**Methods:**

An instrument development multiphase mixed method study (exploratory sequential) was conducted in two phases: item development and qualitative content validation followed by validation of the instrument. Feedback was taken from 15 experts for qualitative content validation in phase 1. In phase 2, content validity was established through three rounds of modified Delphi. Validity evidence was collected for the content (content validity index), response process (cognitive interviews), internal structure (confirmatory factor analysis), and internal consistency (Cronbach’s alpha).

**Results:**

The 48-item preliminary instrument was reduced to a 28-item instrument with eight domains: self-anonymity, privacy settings, maintenance of boundaries and confidentiality, conflict of interest, accountability, respect for colleagues, and ethics. The content validity index of the scale was 0.91. The reliability and construct validity of the instrument was established by responses from 500 healthcare professionals from multiple hospitals. Confirmatory factor analysis showed a model with a goodness-of-fit index of 0.86, root mean square error of approximation of 0.06, and observed normed χ^2^ of 2.7. The internal consistency through Cronbach's alpha α was 0.96.

**Conclusion:**

The digital professionalism self-assessment instrument (DP-SAI) has an appropriate level of content and measures the construct reliably. It can be used by medical doctors, dental clinicians, nurses, physiotherapists, and clinical pharmacists to self-assess and reflect on their social media practices. This will help to address these issues to enhance the quality of online communication through various social media platforms.

**Supplementary Information:**

The online version contains supplementary material available at 10.1186/s12909-024-05142-6.

## Introduction

Social media (SM), frequently referred to as Web 2.0, encompasses digital technologies that aid the development and sharing of ideas, content, and other forms of expression via networks and virtual communities [1]. The use of social media in the healthcare sector has surged exponentially as more professionals are joining the digital realm to communicate and network professionally [2]. The COVID-19 pandemic further escalated its use for consultations and the rapid dissemination of new knowledge [3]. In a time of social distancing, lockdown, and travel restrictions, these sites facilitated easy communication between people across the globe so much that by mid-2020, 3.96 billion people, more than half of the world’s population, became active social media users [4].

The ease and speed with which professional healthcare can be accessed and sought through these web-based applications is undeniable, but it has posed new challenges of unprofessional conduct and controversial posts regarding healthcare. Information technology experts have been warning the public throughout the digital revolution that nothing in any form shared through digital technology is guaranteed to stay safe and private [5]. Healthcare Professionals (HCPs) disclosing patients’ information for the sake of consultation with peers and senior colleagues violate patients’ privacy and confidentiality, as they are not fully aware of the implications of these online practices owing to a lack of training and assessment of ethical digital communication [6].

This led to the emergence of a new dimension of professionalism: e-professionalism/online professionalism/digital professionalism. E-professionalism has been defined as “the attitudes and behaviours reflecting traditional professionalism paradigms but manifested through digital media” [7]. Understanding this form of professionalism is essential to incorporate it as a competency for HCPs. Many theories have been proposed to understand digital professionalism. Petronio et al.’s communication privacy management theory states that effective privacy management is essential to balance the disclosure of information, private ownership, boundaries, and control. They proposed that by sustaining a stringent privacy regulation process, HCPs can maintain a professional physician‒patient boundary while safeguarding their privacy. Privacy settings and maintaining virtual boundaries conform to ownership and control over physicians’ personal information while respecting the privacy of patients as well [8]. When patients share information with HCPs, they become confidants and co-owners of information, and thus, maintaining confidentiality becomes essential to avoid privacy turbulence.

Another related theory explaining the ethical and behavioral aspects of digital professionalism is Azjen’s theory of planned behavior. Being accountable for one’s actions, declaring a conflict of interest, and respecting colleagues and ethical practice depend on digital norms and attitudes towards the use of social media, and the ability to exercise control while interacting digitally with patients [9].

Considering how the unethical use of social media can strain the social contract between medicine and society, there is a growing need to develop assessment principles, criteria, and valid instruments to assess HCPs’ social media attitudes and behaviour [10]. The healthcare authorities and regulatory bodies have issued professional standards, guidelines, evidence-based reports, and consensus statements [11]. The literature showed three scales/ questionnaires, related to online professionalism. These scales and questionnaires are for students and are not appropriate to be used in the context of HCPs. The medical students use social media mainly for educational purposes, and they are more interested in learning than giving patient advice. These tools for social media use are either too specific, focusing on the class or campus setting and the quality of information shared [12, 13], or too general, covering the whole of cyberspace [14]. Therefore, there was a need for a tool that could specifically evaluate the online behaviour of HCPs regarding patient and professional advice. Self-assessment is part of the continuous learning process of adult learners and promotes personal accountability. A self-assessment tool will help HCPs identify their areas of improvement regarding online presence and communication and will provide them with guidance on enhancing their online credibility by avoiding potential pitfalls. It will also help them reflect on their online behaviour and align their online image with their personal and professional goals.

Due to globalisation and the diversity of the world, HCPs interact with a wide variety of patients from diverse cultures and ethnic groups on digital platforms; a tool that helps them review their biases and assumptions and render them more culturally sensitive is an absolute necessity. A tool addressing various aspects of digital professionalism can foster a common understanding of online conduct, promote positive interactions, and minimise the risk of misunderstandings or conflicts arising from cultural differences.

Multiple documents related to SM guidelines by universities, medical boards, and accrediting bodies are available, and they have outlined almost similar domains of digital professionalism. Of these guidelines, the General Medical Council (GMC) [15] and General Dental Council (GDC) [16] of the UK have outlined detailed guidelines in their document “Ethical Guidance for doctors.” The doctor’s use of social media was published in 2013 as an extension to address digital conduct on social media networks and encompasses the essential aspects of this form of professionalism. Thus, GMC domains were used as they were the most comprehensive, encompassing all major areas of digital professionalism. These guidelines are evidence-based and are constantly updated based on the evolving trends of the digital world.

**Fig. 1:**
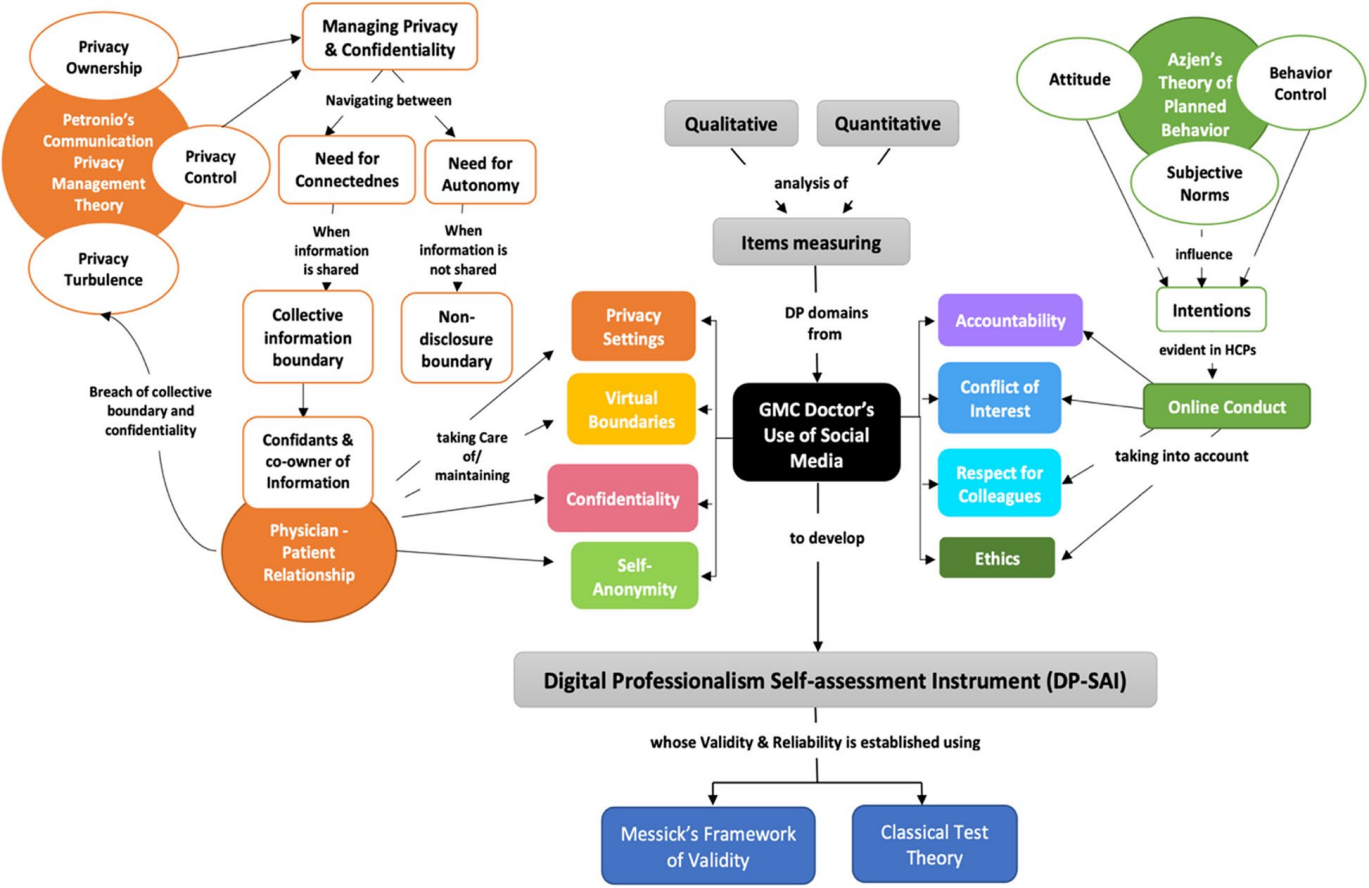
Conceptual framework for development of digital professionalism self-assessment instrument

Figure 1 shows a conceptual framework that was designed incorporating domains of digital professionalism, Petronio’s communication privacy management theory, and Azjen’s theory of planned behaviour. This framework helped to understand the construct and the item development process, served as a blueprint for methodology, and helped in answering the following research questions: 1) What are the key domains and items that adequately assess the domains of digital professionalism of healthcare professionals using social media? 2) How can a self-assessment instrument assessing the digital professionalism of HCPs using social media be validated?

## Methods

**Fig. 2:**
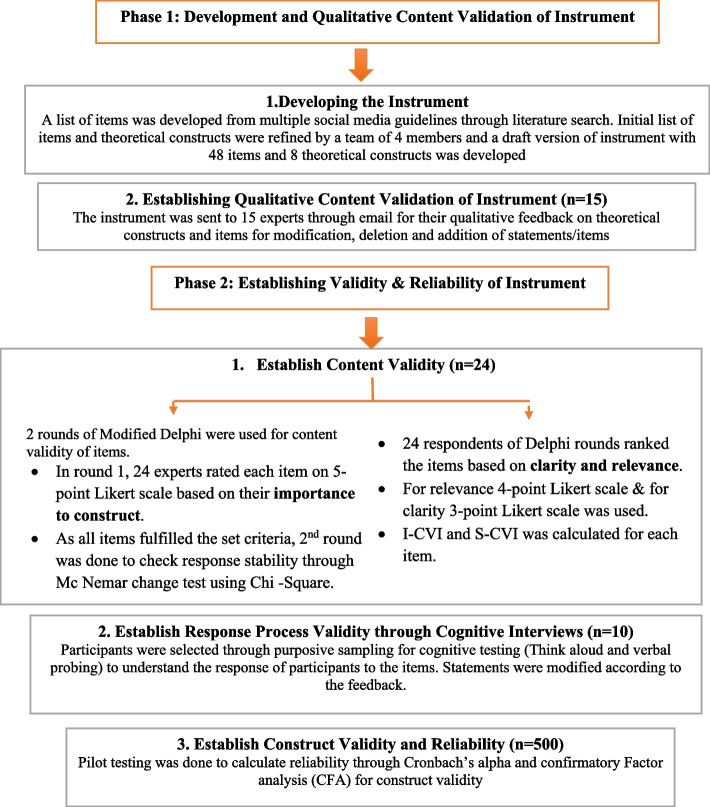
Phases of the study that show the development and validation of the self-assessment instrument measuring the digital professionalism of HCPs

The study was conducted in two phases using the instrument development multiphase mixed-method design (exploratory sequential) from February 2022 to July 2022 (Fig. 2). Ethical approval was obtained from Riphah International University (Riphah/IIMC/IRC/22/2001) and Islamabad Medical and Dental College, Pakistan (No. 56/IMDCIIRB-2022). The participants were HCPs (medical doctors, dental clinicians, nurses, physiotherapists, speech therapists, clinical and community pharmacists). Written informed consent was obtained from all participants during various phases of the study.

### Phase 1- Instrument development and qualitative content validation

Table 1 shows the eight domains of digital professionalism that were identified from GMC social media guidelines. The items were constructed using multiple social media guidelines as shown in Table 2. The guidelines were searched using PubMed, ERIC, BioMed Central and Google Scholar. Only full text, freely accessible guidelines regarding online/digital professionalism of HCPs (medical and allied healthcare professionals) were included while those for undergraduate medical, dental, and allied sciences students were excluded.

**Table 1:** Constructs of digital professionalism and their definitions

Constructs	Definitions
**1. Privacy settings**	Part of social networking website or internet browser that allows one to control who can have access to personal information or see the content posted
**2. Self-anonymity**	A condition in which the identity of healthcare professionals is not known to others
**3. Maintaining Confidentiality**	Limit the disclosure of a patient’s identity and any data entrusted to professionals during assessment, diagnosis, and treatment and protect it against unauthorized access
**4. Maintaining Boundaries**	Ability to recognize and draw a line between a professional and a personal relationship
**5. Conflict of Interest**	A situation in which a healthcare professional is at risk of acting in a biased way because of personal, commercial, or financial interests
**6. Accountability**	Extent to which healthcare professionals are answerable to patients, colleagues, employers and society for their behaviour, judgement, and decisions
**7. Respect for Colleagues**	Treating co-workers and colleagues with respect, kindness, courtesy, and politeness
**8. Ethics**	Moral principles that govern a person’s behaviour or the conduct of an activity

**Table 2:** The governing bodies and the documents analysed

Governing Body	Document Name
**1. General Medical Council (GMC) (UK)**	Doctor’s use of social media [15]
**2. General Dental Council (GDC) (UK)**	Guidance on using social media [16]
**3. American Medical Association (AMA)**	AMA policy: professionalism in the use of social media [17]
**4. British Medical Association (BMA)**	Social media, ethics and professionalism BMA guidance [18]
**5. World Medical Association (WMA)**	WMA statement on the professional and ethical use of social media [19]
**6. American Nurse Association (ANA)**	ANA's Principles for Social Networking and the Nurse: Guidance for Registered Nurses [20].
**7. Australian Medical Association and New Zealand Medical Association**	Social Media and the Medical Profession: A Guide to Online Professionalism for Medical Practitioners and Medical Students [21].
**8. College of Physiotherapists of Ontario**	Social media principles for Physiotherapists

Items written in statement form were matched to response anchors with a 5-point Likert scale. The first version of the instrument was emailed to 15 experts, including HCPs and medical educationists with five years of experience for modification, deletion, and addition of items. Experts’ feedback was analysed, and changes were made based on the following criteria: (1) relevance of the item to construct, (2) ease of understanding, (3) removal of duplicate or ambiguous items, and (4) elimination of spelling and grammatical errors [22].

### Phase 2: Instrument validation

#### Content Validity

Content validity was established through a) the consensus-building modified Delphi technique and b) the content validity index (CVI). Thirty-five national & international experts were selected based on the following criteria: HCPs who had worked on digital professionalism and/or professionalism and medical educationists with master’s degrees or above with more than five years of experience.

##### Modified Delphi Round 1

The content validation Google forms were emailed to 35 experts. They included a summary of the project and informed consent. Moreover, each domain was defined to facilitate scoring along with a short video explaining the instructions. The experts were requested to rank each item based on its importance in measuring the construct on a 5-point Likert scale (very important = 5, important = 4, moderately important = 3, less important = 2, and unimportant = 1). An open-ended question was included at the end of every section of the instrument, and the participants were requested to justify the extreme options.

##### Data Analysis

Data were analysed using SPSS version 26. The median and interquartile ranges (IQRs) were calculated for each item. The criteria for the acceptability of an item in Delphi rounds were decided beforehand [23];

- Agreement of ≥ 75% of the experts on the upper two measures (very important or important)
- Median score of ≥ 4
- An interquartile range of ≤ 1 on a 5-point Likert scale

##### Modified Delphi Round 2

The forms in Word format with percentage agreement of all participants on very important and important, median, and IQR, and the response of the expert in the previous round were emailed individually to the respondents of round 1. Stability refers to the consistency of responses and is established if the responses obtained in two successive rounds do not significantly differ from each other [24]. Experts were requested to review their responses in round 1 and to rank the items again on the previous scale if they wanted to change them.

##### Data Analysis

Data were analysed using SPSS 26, and stability was calculated through the McNemar change test using nonparametric chi-square statistics to calculate the p value of each item [25, 26]. The value was set at 0.05.

##### Modified Delphi Round 3

Google forms were emailed to respondents of previous rounds, who were requested to rate each item on a 4-point Likert scale on relevance (highly relevant = 4, quite relevant = 3, somewhat relevant = 2, and not relevant = 1) and a 3-point Likert scale on clarity of the items (very clear = 3, item needs revision = 2, and not clear = 1).

##### Data Analysis

The ratings of 3 or 4 on the relevance scale were recorded as “1”, and items ranked 1 or 2 were recorded as “0”. The content validity index of individual items (I-CVI) was calculated by adding 1 s for each item and dividing by the total number of experts (*n* = 24) [22]. The average CVI scores across all the items gave the content validity index of scale (S-CVI) [27, 28]. Items having an I-CVI of ≥ 0.90 were included. Those between 0.78 and 0.90 were revised, and items with I-CVI ≤ 0.78 were removed [22]. The content clarity average (CCA) was calculated, and items with CCA values above 2.4 (80%) were marked as very clear [22].

#### Response process validity

Cognitive pretesting of the instrument was performed through in-person semi-structured interviews of ten participants using convenience sampling. Pilot testing was performed to identify and resolve any potential issues. Think-aloud and verbal-probing techniques were used with concurrent probes. Notes were taken by the researcher during interviews, which were also audio recorded after taking the participants’s consent for later analysis.

##### Data analysis

Audiotaped interviews were transcribed and segmented. Analytic memos were created and coded using predefined categories: (1) items with no problems, (2) with minor problems, and (3) with major problems [29]. This coding was performed by two co-authors independently to assure inter-rater reliability. Moreover, the principal author analysed the coding to solve any differences.

#### Pilot testing

Piloting was performed to establish the construct validity and internal consistency of the instrument. Many criteria are used to calculate the sample size of pilot testing, such as a subject-to-variable ratio (SVT) of 10:1 [30] and ranges: N ≥ 1000 is excellent, ≥ 500 is good, 100–500 is fair, and < 100 is poor for factor analysis [31], where N is the number of participants. However, a larger sample size decreases sampling error, and it must increase with an increase in the number of factors [32]. Thus, for this study, a sample size of 550 was used for pilot testing and factor analysis, and participants were emailed Google forms. Reminders were sent on Day 5 and Day 10 through email and WhatsApp to increase the response rate.

##### Data analysis

Data were analysed by SPSS for descriptive statistics and internal consistency. Construct validity was established through confirmatory factor analysis (CFA) using Analysis of Moment Structure (AMOS) 24.0. Exploratory factor analysis (EFA) was not performed, as there were specific expectations regarding (a) the number of constructs or factors, (b) which items or variables reflect given factors, and (c) whether the factors or constructs were correlated [33]. EFA is performed when the factors are not known or are yet to be determined. While CFA is preferred when there is a strong model based on past evidence regarding the number of factors and which items are related to which factors. The GMC guidelines are comprehensive, evidence-based, and constantly updated based on new research and rapidly evolving digital norms and trends. Thus, the domains of digital professionalism from “Doctors use of social media” by GMC were used, and CFA was done to examine the latent structure and item-factor relationship [34].

None of the items was reverse coded. While entering the data in SPSS, all the items were considered as continuous variables, as all were on the same Likert scale, and the choices were taken as “Always, Usually, About half the time, Seldom, and Never” from 5 to 1, respectively.

## Results

### Phase 1: Instrument development and qualitative content validation

Eight constructs measured by 48 items were identified from social media guidelines. A total of 15 participants (RR = 100%) responded, and 40 items were selected after modification and deletion based on their feedback (see Additional file 1: Appendix A).

### Phase 2: Instrument validation

#### Content validity

A total of 24 experts (*n* = 24/35) responded in Delphi round 1 with a response rate of 69%. All the items met the predefined criteria with a median ≥ 4, IQR ≤ 1, and the combined percentage of the upper two options ≥ 75%. A total of 24 experts (100%) responded in round 2, and all items showed stability with a p value > 0.05, i.e., there was no statistically significant difference between the responses of experts in two consecutive rounds. In round 3, 23 experts (96%) participated. Five items with an I-CVI less than 0.78 were removed, four items with an I-CVI between 0.78–0.90 were modified, and the rest of the items with an I-CVI greater than 0.90 were accepted. Thirty-four items had CCA > 2.4 and were accepted, while six items with CCA < 2.4 were rephrased (see Additional file 1: Appendix B). Thus, the items were reduced to thirty-five at this stage. The average clarity of the scale was 2.8, and the S-CVI/AVG was 0.91.

#### Response process validity

Based on cognitive interviews with 10 participants (*n* = 10), seven items were rephrased to improve clarity, and two items were merged, while two items were deleted due to major problems (see Additional file 1: Appendix C).

#### Pilot testing

**Fig. 3:**
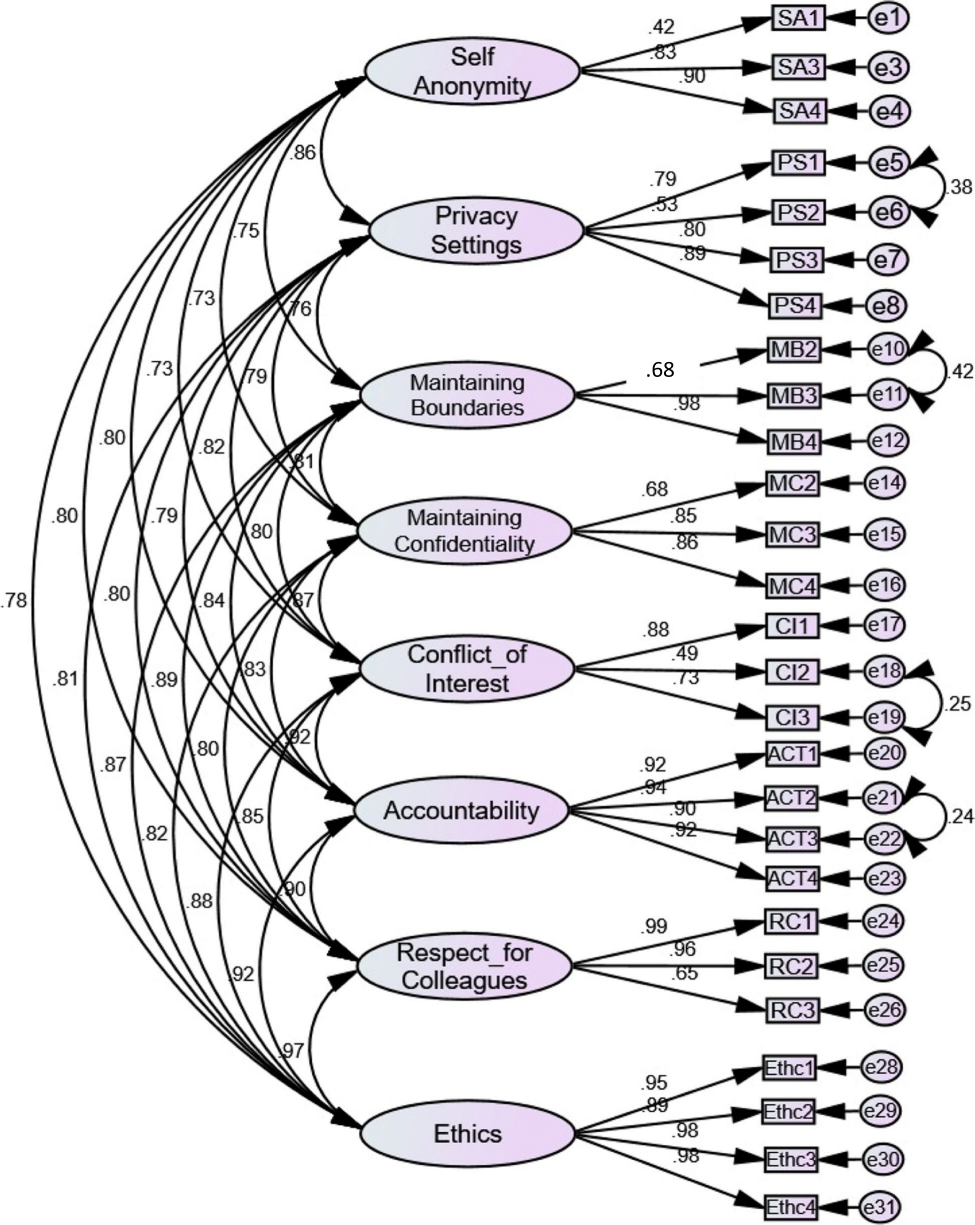
Sequential Equation Model for Instrument. The figure shows factor loadings, factor correlations, and good fit indices (parsimonious, absolute, and incremental fit) for an eight-factor model containing 27 items. Abbreviations used: SA = Self-Anonymity, PS = Privacy Settings, MB = Maintaining Boundaries, MC = Maintaining Confidentiality, CI = Conflict of Interest, ACT = Accountability, RC = Respect for Colleagues, Ethc = Ethic

A total of 500 participants (RR 91%) responded, of whom 210 (42%) responses were obtained on Google forms, while 290 responses (28%) were received on paper-based forms. The results of the Kaiser-Meyer Olkin Measure of Sampling adequacy (KMO) showed an adequate sample for factor analysis (0.962, *p* < 0.01). The CFA resulted in a model with a good fit as shown in Fig. 3. Table 3 shows the goodness-of-fit for the models, reported through Chi Sq/df, RMSEA, CFI, NFI, TLI, GFI, and AGFI with GFI of 0.86, RMSEA of 0.06, and observed normed χ^2^ 0f 2.7.

**Table 3:** Results of confirmatory factor analysis of the 32-item instrument (*n* = 500)

Fit Indices	Cut-off values	Measured values
**1. Incremental Fit Measures**		
• Normed fit index (NFI)	> 0.08 -1 0.09 (good fit model)	0.936
• Incremental fit index (IFI)	Near to 1	0.959
• Relative fit index (RFI)	Near to 1	0.923
• Tucker‒Lewis index (TLI)	Near to 1	0.950
• Comparative fit index (CFI)	Near to 1	0.959
**2. Absolute Fit Measures**		
• Root mean square error of approximation (RMSEA)	0.08–0.1 0.05 (good fit model)	0.065
• Goodness-of-fit index (GFI)	> 0.08–1 0.09 (good fit model)	0.862
• Observed normed χ^2^ (CMIN/df)	< 5	2.73

Four items were dropped for the goodness-of-fit model, as they had low loading values (< 0.40), 2nd item of self-anonymity (SA 2), 1st item of maintaining boundaries (MB 1), 1st item of maintaining confidentiality (MC 1), and 5th item of accountability (ACT5). The residual covariance value of the 4th item of respect for colleagues (RC4) was high (> 2) and was thus deleted. The value of Cronbach's alpha α of the instrument was 0.96, and the subscales ranged from 0.61 – 0.97 as shown in Table 4. Thus, the validation process reduced items from 48 in preliminary draft to 28 items in the final instrument as shown in Table 5.

**Table 4:** Results of reliability analysis of 28 items (*n* = 500)

Items	Scale Mean if Item Deleted	Scale Variance if Item Deleted	Corrected Item-Total Correlation (CITC)	Cronbach's Alpha if Item Deleted (CAID)
**Self-anonymity**				
1. I identify myself by name and profession on publicly accessible social media sites and networks	87.208	1311.736	.471	.969
2. I am cautious when posting my personal information on professional social media platforms	87.174	1277.671	.731	.967
3. I bear in mind that any post uploaded anonymously can, in many cases be traced back to its source or point of origin	87.192	1273.538	.783	.967
**Privacy settings**				
4. I apply conservative/strict privacy settings and carefully select the intended audience on my personal social media profile	87.310	1270.130	.794	.967
5. I regularly review the privacy settings of my personal and professional social media profiles	87.014	1306.415	.535	.968
6. I keep in mind that the privacy settings are imperfect and any content posted online is public and widely accessible	87.206	1281.326	.735	.967
7. I bear in mind that once the information is posted online, it is difficult to remove it completely as users may distribute it further or comment on it	87.290	1270.992	.808	.967
**Maintaining boundaries**				
8. I do not entertain my patient's queries about healthcare if they access me through my private/personal social media profile	86.962	1345.303	.134	.971
9. I avoid establishing online personal contacts with my patients like accepting friend requests	87.162	1278.364	.690	.968
10. I respect the privacy of my patients and do not search their social media profiles	87.298	1248.025	.863	.967
**Maintaining confidentiality**				
11. I avoid posting masked/unidentifiable/anonymized images of my patients on social media sites when informed consent of patients could not be obtained	87.252	1272.758	.684	.968
12. I refrain from discussing my patient's complaints and treatment with colleagues on publicly accessible social media sites	87.214	1270.770	.755	.967
13. I keep in mind that an unnamed patient may be identifiable through minimal information even in a private online forum	87.250	1270.492	.787	.967
**Conflict of interest**				
14. I specify that the opinions I express online are my own and do not reflect another employer, colleague, or institute	87.230	1261.356	.846	.967
15. I declare any financial or commercial conflict of interest when posting content online (health care organizations, pharmaceutical, and biomedical companies)	87.162	1305.375	.496	.969
16. I refrain from endorsing and promoting healthcare products and events on social media sites based on my personal experience	87.328	1279.002	.723	.967
**Accountability**				
17. I keep in mind that the content I post online is subject to the same laws of copyright and defamation as written or verbal communication	87.264	1256.940	.889	.966
18. I acknowledge the original source while posting healthcare-related information and post evidence-based facts on my professional accounts	87.380	1258.809	.884	.966
19. I keep in view the legal implications (defamation, cyberbullying, privacy lawsuits, copyright breach) of my online posts regarding patient care and management	87.262	1262.783	.868	.967
20. I comply with social media guidelines for healthcare professionals while using social media platforms for professional use	87.422	1259.206	.883	.966
21. I keep in mind that any information I share online as a healthcare professional represents the medical profession at large and is trusted by the public	87.340	1254.497	.901	.966
**Respect for colleagues**				
22. I treat my colleagues with respect and do not bully, harass, or post baseless comments about them on social media forums and blogs	87.356	1240.126	.903	.966
23. I keep in mind that my comments on my colleague’s content can negatively affect their reputation	87.358	1244.844	.888	.966
24. If I see unprofessional content posted by my colleague, I feel responsible to bring it to the attention of that person	87.234	1282.869	.727	.967
**Ethics**				
25. I keep my relationship with patients strictly professional and do not exploit them for any personal or financial gains	87.322	1241.698	.899	.966
26. I recognize and resolve ethical issues (e.g., breach of privacy, confidentiality & trust, relationship abuse) encountered during online communication with patients	87.334	1256.067	.880	.966
27. I take care of patient safety and trust while giving medical advice during online interactions	87.404	1247.957	.904	.966
28. I respect the diversity (ethnicity & racial differences) of my patients and colleagues during online interaction	87.376	1244.788	.906	.966

**Table 5:** Modifications performed in the instrument during validation

**Instrument**	**Expert Feedback**	**Content Validity**	**Response process validity**	**Construct validity**	Final Instrument
	**Instrument Version 1**	**Instrument Version 2**	**Instrument Version 3**	**Instrument Version 4**	
**Total items**	**48**	**40**	**35**	**32**	**28**
Items accepted without change	31	30	29	28	----
Items accepted after modification	8	5	7	0	----
Items deleted	8	5	3	4	----
New items added	1	0	0	0	----
**Final items**	**40**	**35**	**32**	**28**	**28**

## Discussion

This study aimed to develop and validate a self-assessment instrument that HCPs can use to assess their online conduct and behaviour through the lens of digital professionalism. The final 28-item instrument showed good content and response process validity. The absolute and incremental fit values of the 8-factor model showed an overall good fit, and its applicability was further strengthened by correlations among the constructs.

Professionalism is culture and context-sensitive and thus there are multiple assessment strategies and tools available to assess this competency at the “does” level of Miller’s pyramid including self-assessment scales, multisource feedback (MSF), entrustable professional activities (EPAs), peer and patient assessments, and comment cards [35]. Most of these tools [29, 30, 36], including the famous Penn State College of Medicine Professionalism Questionnaire (PSCOM) [37], incorporate professionalism domains outlined by the American Board of Internal Medicine (ABIM): accountability, excellence, duty, integrity, altruism, and respect for others [38]. The core professional values might remain the same, but online interaction differs significantly on issues such as privacy settings, data privacy, and professional virtual boundaries. An understanding of the digital professionalism domains is essential for establishing course contents and assessment tools. The major domains identified from the GMC social media guidelines “Doctor’s use of Social Media” [15] are particularly relevant to professionalism while using these sites.

First four of these domains self-anonymity, privacy settings, maintaining boundaries and confidentiality, are related to patient-physician boundaries. According to Petronio’s communication privacy management theory, effective privacy management is essential for a balance between disclosure of information, privacy ownership, boundaries and control. Self-anonymity is related to the extent to which personal information is disclosed on personal and professional social media sites and the digital footprints one leave behind even when something is posted anonymously [39]. Similarly, the next two domain, privacy settings and maintaining virtual boundaries are conforming to the ownership and control over physician’s personal information while respecting privacy of patient as well [8]. When patients share information with healthcare professionals, they both become confidants and co-owner of information and thus maintaining confidentiality becomes essential to avoid privacy turbulence.

Last four domains, conflict of interest, accountability, respect for colleagues, and ethics, relate to Azjen’s theory of planned behaviour. Being accountable for one’s actions, declaring conflict of interest, respecting colleague and ethical practice depends on digital norms, attitude towards use of SM and ability to exercise control while interacting digitally with patients [9].

Similar domains have been highlighted by some recent studies [40–46]. These domains closely resemble major areas of professionalism with a main focus on the digital realm. Moreover, the items were constructed using multiple social media guidelines and thus, the items covered all aspects of digital professionalism for all HCPs to provide a holistic and comprehensive self-evaluation.

The current literature showed three scales/questionnaires of digital professionalism designed for medical students. Marelić et al. developed and validated a scale to assess the attitudes of medical and dental students toward e-professionalism. Mosalanejad and Abdollahifard developed and validated a questionnaire to assess the professionalism of cyber users in medical sciences in Iran. A 15-item scale was developed and validated by Chisholm Burns et al. for assessing the online professionalism of pharmacy students.

These studies used domains from previous studies and extracted factors through exploratory factor analysis (EFA). However, in our study, domains were identified from GMC, and thus, only CFA was performed due to certain expectations regarding the number of factors and their correlations [33]. This approach has been used previously in which CFA was performed to establish the construct validity of the teacher and student questionnaires to explore curriculum viability [22].

During CFA, five items showed weak factor loading and correlations. One of the items, SA 2, “I describe my credentials while expressing my opinion on medical issues in blogs and forums”, showed weak loading, which might be because blogs and forums are among the same professionals, and they are already familiar with each other. Moreover, these forums are used sparingly in our setting, in which WhatsApp and Facebook emerged as the most popular platforms. Although AC item 5, “I keep in mind that any information I share online as a healthcare professional represents the medical profession at large and is trusted by the public,” showed weak loading, this item is important as whatever information HCPs post online is trusted by the public and is usually taken as the opinion of the medical profession at large. The item showed good, corrected item-total correlation (CITC) and was thus retained in the final instrument, as it was important in measuring the accountability domain.

The main strength of our study was the extensive methodology that was followed according to instrument development guidelines reported in the literature. Moreover, reliability and internal consistency were established by taking responses from participants from multiple hospitals and institutes of the country with good representation of doctors, paramedical staff, and clinical pharmacists.

This study was not without limitations. For Delphi rounds, international experts identified from the database were contacted through emails, but the response rate was low. Second, convenience sampling was performed for pilot testing instead of random sampling. Moreover, self-assessment instruments have limitations such as respondent bias and lack of observation.

One of the avenues for potential future investigations is that the validity of the instrument is established on larger random samples from different professional groups, cultures and contexts countrywide and globally. Further research is required to design and implement courses incorporating digital professionalism to train future physicians, dentists, and paramedical staff for safe and professional online communication through social media. We also suggest an evaluation of the outcome of this self-assessment instrument in improving future digital practices of HCPs after training them.

## Conclusion

Social media awareness and familiarity with its use resonates as an essential skill for medical practitioners. Our findings suggest that the 28-item DP-SAI has an appropriate level of content, measures digital professionalism reliably, and represents the target population of HCPs. This is an important advancement in terms of reporting lapses in online conduct and will help in proposing solutions to enhance the quality of online professional communication through SM.

### Supplementary Information

**Additional file 1: Appendix A.** Feedback of experts for qualitative content validation in phase 1. **Appendix B.** Results of modified Delphi Round 1 in phase 2. **Appendix C.** Responses of experts during cognitive pre-testing during response process validation.

## Data Availability

The data generated and analysed during the study are available on request. The corresponding author Shazia Imran can be contacted for the data. (drshaziahassan@gmail.com).

## Abbreviations

HCPs: Healthcare Professionals
SM: Social Media
GMC: General Medical Council
GDC: General Dental Council
AMA: American Medical Association
BMA: British Medical Association
ANA: American Nurse Association
ABIM: American Board of Internal Medicine
