# Supplementary material for: Development and validation of self-assessment instrument to measure the digital professionalism of healthcare professionals using social media
Source: BMC Med Educ. 2024 Mar 6;24:243. doi: 10.1186/s12909-024-05142-6 (PMC10919038; doi:10.1186/s12909-024-05142-6)
Supplement: Supplementary file 1 — Additional file 1: Appendix A. Feedback of experts for qualitative content validation in phase 1. Appendix B. Results of modified Delphi Round 1 in phase 2. Appendix C. Responses of experts during cognitive pre-testing during response process validation. [file 12909_2024_5142_MOESM1_ESM.pdf]

Additional File: 1
Appendix A
Table of Expert Feedback for Qualitative Content Validation in Phase 1

I.	Maintaining Confidentiality	Accept	Modify	Delete	New Item Suggested
1.	I post identifiable patient images on social media sites with the informed consent of patients.				You may add a related item (TS) I post masked/ unidentifiable/anonymized images of patients on social media sites when informed consent of patients could not be obtained.
2.	I discuss my patient's complaints and treatment with colleagues on publicly accessible social media sites.	A			
3.	I keep in mind that an unnamed patient may be identifiable through minimal information even in a private online forum.	A			
4.	I make sure that any personal online information about the patient is effectively protected against improper access, disclosure, or loss.	A			
**II.**	**Maintaining Boundaries**	**Accept**	**Modify**	**Delete**	**New Item Suggested**
5.	I accept a friend request from my patients.		Delete “A” (AT)		
6.	I entertain patient queries about health care if they access me through my private/personal social media profile.	A	“Item # 6 and 9: Entertaining patient queries and giving specific clinical advice to patients appear to have the same meaning”. (RS)		
7.	I use separate social media profiles for personal and professional use.	A			
8.	I avoid establishing online personal contacts with my patients.	A	“Merge items 5 & 8” (HZ) Accepted		

9.	I provide specific clinical advice to patients on social media sites.		I provide clinical advice to anyone who approaches me on social media. (MM) Accepted		
	**Additional comments:** "In my opinion item numbering should be changed, my suggested numbering is 8,7,5,6, and 9 (SM). Accepted<br>"Items 6 and 9 seem overlapping, we can either remove 9 or rephrase it to distinguish it from 6. Item 6 appears to be about patients one is already seeing (in clinic/hospital) etc. while 9 is addressing general population". (MM) (Accepted)				
**III.**	**Conflict of Interest**	**Accept**	**Modify**	**Delete**	**New Item Suggested**
10.	I declare any financial or commercial conflict of interest when posting content online (health care organizations, pharmaceutical, and biomedical companies).	A			
11.	I endorse and promote products, private events, or groups including pharmaceutical companies on social media sites based on my personal experience.		"Add the phrase "online/on social media. The products should be separated from events and in a different question". (TS) Accepted		
	**Additional comments:** "Please write three statements for three different areas of personal, commercial, or financial interests and separate products and events/groups in 11 number questions. Hence there will 05 questions in this theme". (RY) Accepted				
**IV.**	**Anonymity**	**Accept**	**Modify**	**Delete**	**New Item Suggested**
12.	I identify myself by name on publicly accessible social media sites/networks.	A			
13.	I identify myself as a doctor on publicly accessible social media sites/networks.		Repetition of 12 (SM & IT) Accepted		
14.	I bear in mind that the content uploaded anonymously can, in many cases be traced back to its source or point of origin.	A			
15.	I promote myself as a professional on social media forums.		I use social media to promote my professional practice. (MM) Accepted		
16.	I am cautious when posting my personal information on professional social media platforms.	A			
17.	I describe my credentials while expressing my opinion online on medical issues.	A			

	**Additional comments:** "Change the theme to self-anonymity". (FK) Accepted				
**V.**	**Privacy**	**Accept**	**Modify**	**Delete**	**New Item Suggested**
18.	I apply conservative/strict privacy settings on my personal social media profile.	A			
19.	I keep in mind that the privacy settings are imperfect and any content posted online is public and widely accessible.	A			
20.	I recognize the fact that my personal information is embedded within my photographs and other content online.			As not concerned with patient privacy required for professionalism (SM)<br>Deleted	
21.	I regularly review the privacy settings of my personal and professional profiles.	A			
22.	I search and visit my patient's social media profiles.		"Needs elaboration". (SM)<br>Rephrased and moved to maintaining boundaries (AM)		
23.	I acknowledge and accept the potential associated risks of social media.			Not clear<br>Deleted	
24.	I carefully select the intended audience and recipients and restrict access to pre-defined individuals or groups.		Replace "the" with "my" maybe? (AM) Accepted	Repetition of Q1 (IT)<br>Merged with item 18	
25.	I monitor my online presence often on social media. (WMA)		Needs elaboration (SM)<br>Modified		
26.	I respect the privacy of my patients.		Repetition of Item 22 (MM)<br>Moved to maintaining boundaries after merging with 22		
	**Additional comments:** "Change the theme to Privacy Settings". (YQ) Accepted				
**VI.**	**Accountability**	**Accept**	**Modify**	**Delete**	**New Item Suggested**

27.	I keep in mind that the content I post online is subject to the same laws of copyright defamation as written or verbal communication.	A	"Item 27 and 28 can be swapped". (MM) Accepted		
28.	I keep in view the legal implications of my online posts regarding patient care and management.	A			
29.	I provide reference and credit back to the original source while posting healthcare-related information.		Replace with acknowledge? (AM) Accepted		
30.	I comply with appropriate guidelines concerning physician online advertising.	A			
31.	I bear in mind that the content I post online as a healthcare provider is likely to be trusted by the patients.			How can I assure you? (SM) Deleted	
32.	I recognize that the information I share online represents views of the medical profession at large.	A			
**VII.**	**Respect for Colleagues**	**Accept**	**Modify**	**Delete**	**New Item Suggested**
33.	I treat my colleagues fairly and with respect on social media sites and blogs.	A			
34.	I do not bully, harass or post baseless comments about my colleagues on social media forums and blogs.		"Similar to 33". (AM)	Elaboration of Item 33. Its elements can be added to 33. (MM) Accepted,	
35.	I keep in mind that my comments on my colleague's content can negatively affect their reputation.	A			
36.	If I see unprofessional content posted by my colleague, I feel responsible to bring it to the attention of that person.	A	"In item 36 you may add. Privately or publicly". (TS)		
37.	I report the misconduct of my colleague who fails to resolve the issue to relevant authorities.	A			
	**Additional comments:** Item # 36 and 37 can be shifted to professional behavior. (RS)				

VIII.	Professional Behavior	Accept	Modify	Delete	New Item Suggested
38.	I obtain patient consent before ensuing electronic communication with them.	A			
39.	I keep my relationship with patients strictly professional and do not exploit them for any personal or financial gains.			Repetition, addressed above (Dr. Faiza)	
40.	I recognize healthcare-related ethical issues encountered during social media use and online communication with patients.		Delete word "health care: (MM)		
41.	I respond and resolve the ethical issues related to health care on social media effectively.			Merge with 40 (MM) Accepted	
42.	I post evidence-based facts and concise information regarding healthcare on my professional accounts.		Moved to accountability		
43.	I follow social media guidelines for health care professionals while using social medial platforms for professional use.		"Already addressed in accountability section. Remove from either section". (MM) Moved to accountability		
44.	I refrain from passing personal comments during online discussions on medical issues.			Repetition of 34 (AT) deleted	
45.	I specify that the opinions I express online are my own and do not reflect another employer, colleague, or institute.		Already addressed in accountability. (MM) Moved to accountability		
46.	I demonstrate commitment to patient safety during online interaction.		Needs elaboration (SM) Modified		
47.	I respect the diversity, ethnicity, and racial differences of my patients during online interaction.	A			
	**Additional comments:**<br>"Item # 42 can be shifted to the section of "accountability" (SM).<br>"Item # 43 can be shifted to the "Accountability" section". (RS) Accepted, A very well constructed questionnaire covering all aspects (HZ)				

Additional file 1
Appendix B
Table of Results of Modified Delphi Round 1

	**Items**	**E 1**	**E 2**	**E 3**	**E 4**	**E 5**	**E 6**	**E 7**	**E 8**	**E 9**	**E 10**	**E 11**	**E 12**	**E 13**	**E 14**	**E 15**	**E 16**	**E 17**	**E 18**	**E 19**	**E 20**	**E 21**	**E 22**	**E 23**	**I-CVI**	**Decision**
	**Self-Anonymity**																									
1.	I identify myself by name and profession on publicly accessible social media sites/networks.	HR1	QR1	HR1	QR1	HR1	HR1	HR1	QR1	HR1	HR1	HR1	HR1	HR1	HR1	HR1	QR1	QR1	QR1	HR1	HR1	HR1	HR1	HR1	1.00	A
2.	I describe my credentials while expressing my opinion on medical issues in blogs and forums.	SR0	QR1	HR1	QR1	HR1	SR0	HR1	QR1	HR1	HR1	HR1	QR1	HR1	QR1	HR1	HR1	QR1	QR1	HR1	HR1	HR1	HR1	SR0	0.86	AM
3.	I am cautious when posting my personal information on professional social media platforms.	QR1	HR1	HR1	QR1	HR1	HR1	HR1	HR1	QR1	HR1	HR1	HR1	HR1	HR1	HR1	HR1	HR1	HR1	HR1	SR0	HR1	HR1	HR1	0.95	A
4.	I bear in mind that any post uploaded anonymously can, in many cases be traced back to its source or point of origin.	HR1	HR1	HR1	QR1	HR1	HR1	HR1	QR1	HR1	HR1	HR1	HR1	QR1	SR0	HR1	HR1	HR1	QR1	QR1	SR0	HR1	HR1	QR1	0.91	A
	**Privacy Settings**																									
5.	I apply conservative/strict privacy settings and carefully select the intended audience on my personal social media profile.	HR1	QR1	HR1	QR1	HR1	HR1	HR1	QR1	HR1	HR1	HR1	HR1	SR0	HR1	HR1	HR1	HR1	HR1	SR0	HR1	HR1	HR1	SR0	0.86	AM
6.	I regularly review the privacy settings of my personal and professional profiles.	QR1	QR1	HR1	QR1	HR1	HR1	HR1	QR1	HR1	HR1	HR1	HR1	HR1	SR0	HR1	HR1	HR1	QR1	SR0	HR1	HR1	HR1	QR1	0.91	A

7.	I keep in mind that the privacy settings are imperfect and any content posted online is public and widely accessible.	HR1	QR1	HR1	QR1	HR1	HR1	HR1	QR1	QR1	HR1	HR1	HR1	SR0	QR1	HR1	HR1	HR1	HR1	HR1	HR1	HR1	HR1	HR1	0.95	A
8.	I recognize the fact that my personal information is embedded within my photographs and other content online.	QR1	HR1	SR0	QR1	HR1	HR1	HR1	QR1	HR1	HR1	HR1	HR1	SR0	SR0	HR1	HR1	HR1	HR1	SR0	QR1	SR0	SR0	QR1	0.73	D
9.	I monitor my internet presence to ensure that the personal and professional-information on my profiles and content posted about me by others is accurate and appropriate.	HR1	QR1	HR1	QR1	HR1	HR1	HR1	QR1	QR1	HR1	QR1	HR1	SR0	SR0	HR1	HR1	HR1	NR0	SR0	QR1	NR0	NR0	HR1	0.73	D
10.	I bear in mind that once the information is posted online, it is difficult to remove it as users may distribute it further or comment on it.	HR1	HR1	HR1	QR1	HR1	HR1	HR1	QR1	HR1	HR1	HR1	HR1	HR1	HR1	HR1	HR1	HR1	HR1	HR1	QR1	HR1	HR1	HR1	1.00	A
	**Maintaining Boundaries**																									
11.	I use separate social media profiles for personal and professional use.	SR0	HR1	HR1	QR1	HR1	QR1	HR1	QR1	QR1	HR1	HR1	HR1	HR1	SR0	HR1	HR1	HR1	QR1	HR1	QR1	HR1	HR1	HR1	0.91	A
12.	I share medical information & answer general health-related questions on my profile.	QR1	SR0	HR1	QR1	HR1	HR1	HR1	SR0	SR0	HR1	HR1	HR1	SR0	QR1	HR1	HR1	HR1	HR1	SR0	QR1	HR1	SR0	HR1	0.73	D
13.	I provide specific clinical advice to my patients on my professional social media profile only.	QR1	HR1	HR1	QR1	HR1	HR1	HR1	QR1	HR1	QR1	HR1	SR0	HR1	QR1	HR1	HR1	HR1	HR1	HR1	QR1	SR0	NR0	SR0	0.82	AM
14.	I do not entertain my patient's queries about healthcare if they access me through my private/personal social media profile.	QR1	HR1	HR1	QR1	HR1	HR1	HR1	QR1	HR1	HR1	HR1	HR1	SR0	QR1	HR1	HR1	HR1	QR1	HR1	QR1	HR1	HR1	HR1	0.95	A

15.	I avoid establishing online personal contacts with my patients like accepting friend requests.	QR 1	HR 1	HR 1	QR 1	HR 1	HR 1	HR 1	QR 1	HR 1	HR 1	HR 1	HR 1	SR 0	QR 1	HR 1	HR 1	HR 1	HR 1	HR 1	HR 1	HR 1	HR 1	QR 1	0.95	A
16.	I respect the privacy of my patients and do not search their social media profiles.	QR 1	HR 1	HR 1	QR 1	HR 1	HR 1	HR 1	QR 1	HR 1	HR 1	HR 1	HR 1	HR 1	QR 1	HR 1	HR 1	HR 1	HR 1	HR 1	HR 1	HR 1	HR 1	QR 1	1.00	A
	**Maintaining Confidentiality**																									
17.	I post identifiable patient images on social media sites with the informed consent of patients.	QR 1	HR 1	HR 1	QR 1	HR 1	HR 1	HR 1	HR 1	HR 1	HR 1	HR 1	HR 1	SR 0	HR 1	HR 1	HR 1	QR 1	HR 1	HR 1	QR 1	HR 1	HR 1	HR 1	0.95	A
18.	I avoid posting even masked/unidentifiable/ anonymized images of patients on social media sites when informed consent of patients could not be obtained.	QR 1	HR 1	HR 1	QR 1	HR 1	HR 1	HR 1	HR 1	SR 0	HR 1	HR 1	HR 1	SR 0	HR 1	HR 1	HR 1	HR 1	HR 1	HR 1	HR 1	HR 1	HR 1	HR 1	0.91	A
19.	I refrain from discussing my patient's complaints and treatment with colleagues on publicly accessible social media sites.	QR 1	HR 1	HR 1	QR 1	HR 1	HR 1	HR 1	QR 1	SR 0	HR 1	HR 1	HR 1	HR 1	HR 1	HR 1	HR 1	HR 1	HR 1	HR 1	QR 1	HR 1	HR 1	QR 1	0.95	A
20.	I keep in mind that an unnamed patient may be identifiable through minimal information even in a private online forum.	QR 1	HR 1	HR 1	QR 1	HR 1	HR 1	HR 1	HR 1	SR 0	HR 1	HR 1	HR 1	QR 1	SR 0	HR 1	HR 1	HR 1	HR 1	HR 1	QR 1	HR 1	HR 1	QR 1	0.91	A
21.	I make sure that any personal online information about the patient is effectively protected against improper access, disclosure, or loss.	QR 1	HR 1	SR 0	QR 1	SR 0	HR 1	HR 1	HR 1	SR 0	HR 1	HR 1	HR 1	HR 1	SR 0	HR 1	HR 1	HR 1	SR 1	SR 0	SR 0	HR 1	HR 1	QR 1	0.73	D
	**Conflict of Interest**																									

22.	I specify that the opinions I express online are my own and do not reflect another employer, colleague or institute.	QR1	HR1	HR1	QR1	HR1	HR1	HR1	HR1	HR1	HR1	HR1	HR1	HR1	HR1	HR1	HR1	QR1	HR1	HR1	QR1	HR1	HR1	HR1	1.00	A
23.	I declare any financial or commercial conflict of interest when posting content online (health care organizations, pharmaceutical, and biomedical companies)	QR1	HR1	HR1	QR1	HR1	HR1	HR1	HR1	QR1	HR1	HR1	HR1	SR0	SR0	HR1	HR1	HR1	QR1	HR1	QR1	HR1	HR1	HR1	0.91	A
24.	I refrain from endorsing and promoting healthcare-related products and events on social media sites based on my personal experience.	SR0	HR1	HR1	QR1	HR1	HR1	HR1	HR1	QR1	HR1	HR1	HR1	SR0	QR1	HR1	HR1	HR1	HR1	HR1	HR1	HR1	HR1	HR1	0.91	A
25.	I refrain from endorsing and promoting private events and groups including pharmaceutical companies on social media sites.	SR0	HR1	HR1	QR1	HR1	HR1	HR1	HR1	QR1	HR1	SR0	HR1	SR0	QR1	HR1	HR1	HR1	SR0	HR1	SR0	HR1	SR0	HR1	0.73	D
	**Accountability**																									
26.	I keep in mind that the content I post online is subject to the same laws of copyright and defamation as written or verbal communication.	HR1	HR1	HR1	QR1	HR1	HR1	HR1	HR1	QR1	HR1	HR1	HR1	NR0	SR0	HR1	HR1	HR1	HR1	HR1	HR1	HR1	HR1	QR1	0.91	A
27.	I acknowledge the original source while posting healthcare-related information.	HR1	HR1	HR1	QR1	HR1	HR1	HR1	HR1	HR1	QR1	HR1	HR1	SR0	QR1	HR1	HR1	HR1	HR1	HR1	QR1	HR1	HR1	HR1	0.95	A
28.	I keep in view the legal implications of my online posts regarding patient care and management.	HR1	HR1	HR1	QR1	HR1	HR1	HR1	HR1	QR1	HR1	HR1	HR1	SR0	QR1	HR1	HR1	HR1	HR1	HR1	HR1	HR1	HR1	HR1	0.95	A
29.	I comply with social media guidelines for healthcare professionals while using social	HR1	HR1	HR1	QR1	HR1	HR1	HR1	HR1	QR1	HR1	HR1	HR1	SR0	QR1	HR1	HR1	HR1	HR1	HR1	QR1	HR1	HR1	HR1	0.95	A

	medial platforms for professional use.																								
30.	I post evidence-based facts and concise information regarding healthcare on my professional accounts.	HR1	HR1	HR1	QR1	HR1	HR1	HR1	HR1	HR1	QR1	HR1	HR1	QR1	QR1	HR1	HR1	HR1	HR1	HR1	QR1	HR1	HR1	HR1	1.00	A
31.	I recognize that the information I share online represents views of the medical profession at large and is trusted by the public.	HR1	HR1	HR1	QR1	HR1	HR1	HR1	SR0	QR1	HR1	HR1	HR1	QR1	HR1	HR1	HR1	HR1	HR1	HR1	QR1	HR1	SR0	SR0	0.86	AM
	**Respect for Colleagues**																								
32.	I treat my colleagues with respect and do not bully, harass or post baseless comments about them on social media forums and blogs.	HR1	HR1	HR1	QR1	HR1	HR1	HR1	HR1	HR1	HR1	HR1	HR1	HR1	HR1	HR1	HR1	HR1	HR1	QR1	QR1	HR1	HR1	HR1	1.00	A
33.	I keep in mind that my comments on my colleague's content can negatively affect their reputation.	HR1	HR1	HR1	QR1	HR1	HR1	HR1	QR1	HR1	HR1	HR1	HR1	QR1	HR1	HR1	HR1	HR1	SR0	HR1	HR1	HR1	HR1	HR1	0.95	A
34.	If I see unprofessional content posted by my colleague, I feel responsible for bringing it to attention of that person.	HR1	QR1	HR1	QR1	HR1	HR1	HR1	HR1	QR1	QR1	HR1	HR1	SR0	QR1	HR1	HR1	HR1	SR0	HR1	HR1	HR1	HR1	QR1	0.91	the A
35.	I report misconduct of my colleague who fails to resolve the issue to relevant authorities.	QR1	QR1	HR1	QR1	HR1	HR1	HR1	HR1	QR1	QR1	HR1	HR1	SR0	QR1	HR1	QR1	HR1	SR0	HR1	QR1	HR1	HR1	QR1	0.91	A
	**Ethics**																								
36.	I obtain patient consent before ensuing electronic communication with them.	HR1	HR1	HR1	QR1	HR1	QR1	HR1	HR1	HR1	HR1	HR1	HR1	SR0	SR0	HR1	HR1	HR1	HR1	HR1	HR1	HR1	HR1	QR1	0.91	A
37.	I keep my relationship with patients strictly professional and do not exploit them for any personal or financial gains.	HR1	HR1	HR1	QR1	HR1	HR1	HR1	HR1	QR1	HR1	HR1	HR1	HR1	QR1	HR1	HR1	HR1	HR1	HR1	HR1	HR1	HR1	QR1	1.00	A

38.	I recognize and resolve ethical issues encountered during social media use and online communication with patients.	HR 1	HR 1	HR 1	QR 1	HR 1	HR 1	HR 1	HR 1	QR 1	HR 1	HR 1	HR 1	QR 1	QR 1	HR 1	HR 1	HR 1	HR 1	HR 1	HR 1	HR 1	HR 1	QR 1	1.00	A
39.	I take care of patient safety and trust while giving medical advice during online interaction.	HR 1	HR 1	HR 1	QR 1	HR 1	HR 1	HR 1	HR 1	HR 1	HR 1	HR 1	HR 1	QR 1	HR 1	HR 1	HR 1	HR 1	HR 1	HR 1	HR 1	HR 1	HR 1	HR 1	1.00	A
40.	I respect the diversity, ethnicity, and racial differences of my patients and colleagues during online interaction.	HR 1	HR 1	HR 1	QR 1	HR 1	HR 1	HR 1	QR 1	HR 1	HR 1	HR 1	HR 1	HR 1	HR 1	HR 1	HR 1	HR 1	HR 1	HR 1	HR 1	HR 1	HR 1	HR 1	1.00	A

HR= highly relevant, QR= Quite relevant, SR= Somewhat relevant, NR= Not relevant I-CVI= Content validity index of items
A= Accepted, AM= Accepted after Modification, D= Deleted

Additional file 1
Appendix C
Table of Cognitive pre-testing for Response Process Validity

Sr. No	Main Constructs	Items	Qualitative Analysis	Codes
1.	Self-Anonymity	I identify myself by name and profession on publicly accessible social media sites & networks.	"I understand this item and I always mention my name and profession on the professional social media sites that I use for interacting with patients and if my post is related to healthcare."	No change
2.		I describe my credentials (qualifications, designation, and /or years of experience) while expressing my opinion on medical issues in blogs and forums.	"I well understand this statement, but I do not always express my degrees especially in informal discussions."	No change
3.		I am cautious when posting my personal information on professional social media platforms.	"I've understood this item and I seldom share my personal information on sites that I use for patient interaction. Platforms mean sites and networks as in item 1, so better change it here too."	Minor problem- Rephrase<br>I am cautious when posting my personal information on professional social media sites and networks.
4.		I bear in mind that any post or content uploaded even anonymously can, in many cases be traced back to its source or point of origin.	"Yes, that I know, and this is something known about social media that people can trace the origin of any post even if it is without a name."	No change
5.	Privacy Settings	I apply conservative/strict privacy settings and carefully select the intended audience on my personal social media profile.	"Yes, I am very choosy about that, and my strict privacy settings are very strict."	No change
6.		I regularly review the privacy settings of my personal and professional profiles.	"I understand this statement but once I've done them, usually I don't recheck them and consider that my settings are in place."	No change
7.		I keep in mind that the privacy settings are imperfect, and any content posted online is public and widely accessible.	"Yes, this is true as content shared or posted online can be shared by others and that is insecurity about online content."	No change
8.		I bear in mind that once the information is posted online, it is difficult to remove it as users may distribute it further or comment on it.	"I understand this item and that once the content is posted online, it is gone, and I have no control over it. It is difficult to remove as people share it further. Just add the word completely to make it clearer."	Minor problem- Rephrase<br>I bear in mind that once the information is posted online, it is difficult to remove it

				completely as users may distribute it further or comment on it.
9.	**Maintaining Boundaries**	I use separate social media profiles for personal and professional use.	"I understand this item and no, I don't have separate profiles for personal and professional use."	No change
10.		I provide specific clinical advice to my patients on my professional social media profile.	"I use only one profile and give my patients advice on it as it reduces the travel time and hassle, This item is not clear and seems to be a repetition of the next item".	Major problem-Removed
11.		I do not entertain my patient's queries about healthcare if they access me through my private/personal social media profile.	"I understand this item that I should not reply to patients if they ask for my advice through a personal profile. I do interact with patients on my personal profile as that is the only one, I have."	No change
12.		I avoid establishing online personal contacts with my patients like accepting friend requests.	"This item is very clear, and I do not accept friend requests from patients, especially on Facebook."	No change
13.		I respect the privacy of my patients and do not search their social media profiles.	"I understand this item and since I do not make friends with the patient, there is no point searching them or their profiles on social media."	No change
14.	**Maintaining Confidentiality**	I post identifiable patient images on social media sites only with the informed consent of patients.	"I have never posted any image of my patient on social media sites even with their consent."	No change
15.		I avoid posting masked/unidentifiable/anonymized images of my patients on social media sites when informed consent of patients could not be obtained.	"Yes, that's true that it is unethical to share even masked images of the patient when patient consent cannot be obtained, and I always avoid that."	No change
16.		I refrain from discussing my patient's complaints and treatment with colleagues on publicly accessible social media sites.	"I understand this item, but I do discuss my patient complaints in groups with my colleagues, maintaining the anonymity of the patient."	No change
17.		I keep in mind that an unnamed patient may be identifiable through minimal information even in a private online forum.	"The way I discuss my patients in private forums is not identifiable but yes patients and similar patients can relate to the information."	No change
18.	**Conflict of Interest**	I specify that the opinions I express online are my own and do not reflect another employer, colleague, or institute.	"This is clear, and I understand that the opinions I give on WhatsApp groups can be attributed to my organization unless I specify it."	No change
19.		I declare any financial or commercial conflict of interest when posting content online (health care	"This is clear, and I seldom do this as I never post content that can have a conflict of interest. I take care of it more while writing an article."	No change

		organizations, pharmaceutical, and biomedical companies)		
20.		I refrain from endorsing and promoting healthcare products and events on social media sites based on my personal opinion and experience.	"This statement is clear and I do not refrain from it and if I find something good, I endorse it."	No change
21.	**Accountability**	I keep in mind that the content I post online is subject to the same laws of copyright and defamation as written or verbal communication.	"I know that the online content is subject to laws of copyright and defamation cases can be filed based on it. My sensitivity for this aspect is more for research articles."	No change
22.		I acknowledge the original source while posting healthcare-related information.	"No, I do not always do that as I do post content from a book or journal in informal groups and don't give reference."	Minor problems- Merged with item 25
23.		I keep in view the legal implications of my online posts regarding patient care and management.	"Yes, I understand the legal consequences of my online posts and I am especially cautious when I advise my patient that it is clear (dose and duration) because if a patient gets harmed, he may act in a legal way for retributions. Legal implications can be explained here for better understanding."	Minor problems- Rephrase<br>I keep in view the legal implications (defamation, cyberbullying, privacy lawsuits, copyright breach) of my online posts regarding patient care and management.
24.		I comply with social media guidelines for healthcare professionals while using social media platforms for professional use.	"This is clear and I do not know of any social media guidelines for using social media so I do not follow any."	No change
25.		I post evidence-based facts and concise information regarding healthcare on my professional accounts.	"This item seems to be repetition of previous item 22 as original source and evidence based refers to closely related concepts."	Minor problems- Merged with item 23
26.		I recognize that the information I share online represents views of the medical profession at large and is trusted by the public.	"This is not very clear, If it can be rephrased that any information that I share as a doctor does represent my profession."	Minor problems- Rephrase<br>I keep in mind that any information I share online as a healthcare professional represents the medical profession at large and is trusted by the public.

27.	**Respect for Colleagues**	I treat my colleagues with respect and do not bully, harass or post baseless comments about them on social media forums and blogs.	"This item is clear enough and I never treat my colleagues with disrespect or post bad remarks on their posts in informal WhatsApp groups that I am part of."	No change
28.		I keep in mind that my comments on my colleague's content can negatively affect their reputation.	"Yes, I understand that whatever I post online can affect my colleague's reputation and I am always cautious about it."	No change
29.		If I see unprofessional content posted by my colleague, I feel responsible to bring it to the attention of that person.	"The statement is clear, and I have done this a few times by messaging my friend and colleague personally about her unprofessional post online."	No change
30.		I report the misconduct of my colleague who fails to resolve the issue to relevant authorities.	"This item needs some clarity as to who I should report the misconduct to, I have never done that as I don't know of any authority that I should report to."	Minor problems- Rephrase<br>I report the misconduct of my colleague, who fail to resolve the issue, to relevant authorities (administrations of the organization or social media, cybercrime complaint centers).
31.	**Ethics**	I obtain patient consent before ensuing electronic communication with them.	"This is somewhat irrelevant as it's always the patient who starts communication and his initiation is a sort of consent."	Major problem-<br>Removed
32.		I keep my relationship with patients strictly professional and do not exploit them for any personal or financial gains.	"Item is clear enough and I never use my relationship with the patient for any other purpose other than medical advice like money or personal benefit."	No change
33.		I recognize and resolve ethical issues encountered during social media use and online communication with patients.	"Ethical issues need to be elaborated here as respondent can think of multiple issues that might not be related to online professionalism."	Minor problems- Rephrase<br>I recognize and resolve ethical issues (e.g., breach of privacy, confidentiality, trust, relationship abuse) encountered during social media use and online communication with patients.
34.		I take care of patient safety and trust while giving medical advice during online interaction.	"Yes, this is clear, and I do take care of the safety of my patient when I am advising treatment or medicines online."	No change

35.		I respect the diversity, ethnicity, and racial differences of my patients and colleagues during online interaction.	"I do understand what ethnicity and racial differences mean. A patient comes from different religions, races, and cultures Diversity needs to be explained in this statement."	Minor problems- Rephrase<br>I respect the diversity (ethnicity & racial differences) of my patients and colleagues during online interaction.
